# Genetic variation in the *HLA-G* 3′UTR 14–bp insertion/deletion and the associated cancer risk: evidence from 25 case–control studies

**DOI:** 10.1042/BSR20181991

**Published:** 2019-05-10

**Authors:** You Jiang, Jun Lu, Yue-E Wu, Xin Zhao, Liang Li

**Affiliations:** 1 Department of General Surgery, Hefei Second People’s Hospital, Anhui Medical University, Hefei 230011, Anhui, China; 2 Department of Electrocardiogram Diagnosis, The Second Affiliated Hospital, Anhui Medical University, Hefei 230060, Anhui, China; 3 Department of Pathology, The First Affiliated Hospital, Anhui Medical University, Hefei 230022, Anhui, China

**Keywords:** Cancer, Human leukocyte antigen-G, Meta-analysis, Polymorphism

## Abstract

Human leucocyte antigen-G (HLA-G) plays an important role in the progression of human cancers. A growing number of published studies have investigated the correlation between the *HLA-G* 3′ untranslated region (3′UTR) 14-bp insertion/deletion (*Ins/Del*) polymorphism and the associated cancer risk in different populations. However, results from previous studies are inconclusive and inconsistent for the different type of cancers. Therefore, we undertook a meta-analysis to assess the effects of the *HLA-G* 14-bp *Ins/Del* polymorphism on cancer risk. A systematic literature search was conducted in PubMed, Web of Science, CNKI, VIP, and Wanfang databases to obtain relevant studies up to 28 January 2019. The pooled odds ratios (ORs) and corresponding 95% confidence intervals (CIs) were used. Twenty-five published case–control studies comprising 4981 cases and 6391 controls were included in the current meta-analysis. The results of the overall analysis revealed that the *HLA–G* 14–bp *Ins/Ins* genotype and *Ins* allele were associated with the total cancer risk in the homozygote comparison model (*Ins/Ins vs. Del/Del*: OR = 0.80, CI = 0.64–1.00; *P*=0.049) and the allelic comparison model (*Ins vs. Del*: OR = 0.89, CI = 0.81–0.99; *P*=0.035), with a protective role. Further subgroup analyses indicated that the *HLA–G* 14–bp *Ins/Del* polymorphism was associated with the risk of breast cancer and oesophageal cancer (EC), and significant risk of cancer was also observed in Mixed populations and population-based (PB). The results of our meta-analysis show that the *HLA–G* 14-bp *Ins/Del* polymorphism plays an important role in cancer risk, particularly in breast cancer and esophageal cancer in Mixed populations. Additional case–control studies with different types of cancer spanning different ethnicities are needed to extend the present findings.

## Introduction

The incidence and mortality of cancer are increasing worldwide, and cancer has been a major human health problem that creates a large economic burden in both developed and undeveloped countries. According to reported statistics, there were approximately 1688780 new cancer diagnoses, and 600920 cases resulting in mortality due to malignant tumours in the United States in the year 2017 [1]. In 2015, there were nearly 4292000 new cancer diagnoses and 2814000 cancer-related deaths in China [2]. Although the underlying mechanism of carcinogenesis is not completely deciphered, a number of studies have demonstrated that the occurrence of cancer is a complicated process, which includes various environmental factors and genetic susceptibilities [3]. Accumulating evidence has shown that individual genetic susceptibility plays a significant role in the occurrence of a tumour. Moreover, the relationship between polymorphisms and cancer risk has been confirmed for many genes [4,5]. Several lines of evidence have indicated that the progression of a tumour could be related to immunoevasion. Human leucocyte antigen (HLA) may play a critical role in the development and progression of cancer by mediating immune responses [6].

HLA-G, a non-classical HLA class I molecule, is known for its suppressive function and has seven different isoforms. Of the seven isoforms, four have membrane-bound forms (HLA-G1 to HLA-G4) and three have soluble forms (HLA-G5, HLA-G6, and HLA-G7) [7]. Differing from the classic HLA class I molecules, HLA-G is characterised by its restricted tissue distribution, low rate of polymorphism, and immunosuppressive properties [8]. The aberrant expression of HLA-G has been considered a mechanism in a wide variety of tumours that helps the tumour cells escape immunosurveillance [9]. HLA-G has been shown to act as a negative regulator of the human immune response by several mechanisms, including the inhibition of the cytotoxic effects of T lymphocytes and natural killer (NK) cells, as well as the prevention of antigen recognition and anti-proliferative responses of CD4^+^ T cells [10]. Accumulating evidence has shown that HLA-G is highly expressed in a variety of tumour tissues, including breast cancer [11], cervical cancer [12], hepatocellular carcinoma (HCC) [13], oesophageal carcinoma (EC) [14], thyroid carcinoma [15], lung cancer [14], gastric cancer [14], colorectal cancer (CRC) [14], and renal cell carcinoma [16]. These studies show that HLA-G may play a pivotal role in the occurrence and progression of malignant tumours.

The human *HLA-G* gene, comprising eight exons and seven introns, is located on chromosome 6p21.3. Several published studies have indicated that some polymorphisms of the *HLA-G* gene are related to cancer development [17]. The 14-bp insertion/deletion (*Ins/Del*) polymorphism in exon 8 of the 3′ untranslated region (3′UTR) of *HLA-G* is the most widely studied polymorphism. It has been demonstrated that the *HLA-G* 3′UTR 14-bp *Ins/Del* variation implicates the stability and isoform splicing patterns of *HLA-G* mRNA [18]. The *Ins* allele is associated with the decreased expression of HLA-G, while the *Del* allele is associated with the increased expression of HLA-G [19]. After Castelli et al. [20] first assessed the correlation between the *HLA-G* 14-bp *Ins/Del* variation and bladder cancer in 2008, a growing number of molecular epidemiological case–control studies have been carried out in different populations to investigate the association of the *HLA-G* 14-bp *Ins/Del* variant with different types of cancers [11,13,21–24]. However, the results of the published articles varied and even contradicted each other. To identify these findings, four meta-analyses of the association between the *HLA-G* 14-bp *Ins/Del* variation and cancer risk were carried out several years ago [25–28]. Although all four meta-analyses reached the same conclusion, that there was no relationship between the *HLA-G* 14-bp *Ins/Del* polymorphism and the risk of overall cancer, the results of their stratified analyses were inconsistent. Due to the relatively small sample sizes included in the previous meta-analyses, all these meta-analyses lacked sufficient statistical power. Since these reports, many new case–control studies have explored the correlation between the *HLA-G* 14-bp *Ins/Del* polymorphism and the risk of different types of cancer; however, the results of these subsequent studies were still inconclusive. Therefore, an updated meta-analysis including all of the currently identified studies was performed to explore the precise association of the *HLA-G* 14-bp *Ins/Del* polymorphism with cancer susceptibility.

## Materials and methods

### Search strategy

A systematic literature search with no language limitation was conducted in PubMed, Web of Science, CNKI, VIP, and Wanfang databases to obtain all eligible studies published before 28 January 2019. The relevant search keywords included: (HLA-G OR ‘Human leukocyte antigen-G’) AND (mutation OR polymorphism OR genotype OR variation) AND (carcinoma OR cancer OR malignancy OR adenocarcinoma OR neoplasm OR neoplasia OR tumour OR tumour). In addition, other relevant articles were acquired by searching the reference lists of the reviews and studies selected from the search parameters described above.

### Inclusion and exclusion criteria

Published articles fulfilling the following criteria were included: (i) articles published in English or Chinese; (ii) studies that evaluated the correlation between *HLA-G* 14-bp *Ins/Del* polymorphism and cancer risk; (iii) studies that designed as case–control or cohort studies; and (iv) studies that contained sufficient data for genotype distribution estimation or the overall odds ratio (ORs) and 95% confidence intervals (CIs). Articles were excluded based on the following criteria: (i) case reports, not case–control studies, letters, comment articles, reviews or meta-analyses; (ii) lacking sufficient data; and (iii) duplicated publications or samples.

### Data extraction

Two investigators (Y.J. and J.L.) independently collected data from the eligible articles in accordance with the inclusion criteria above. Data extracted from all of the selected studies included the following information: the first author, publication year, country, study population ethnicity, cancer type, sources of controls, genotyping method, number of cases and controls for the 14-bp *Ins/Del* genotypes of *HLA-G*, and results of the Hardy–Weinberg equilibrium (HWE) test in controls. In cases of inconsistent evaluations, all investigators were consulted to obtain a consensus of inclusion or exclusion of the study in the present meta-analysis.

### Methodological quality assessment

The quality of the included studies was appraised according to the Newcastle–Ottawa Scale (NOS) by two independent investigators. Each study had a calculated score based on three criteria including selection, comparability, and exposure (maximum score = 9 points). The score of a study must be higher than 5 to be included in the present meta-analysis (http://www.ohri.ca/programs/clinical_epidemiology/oxford.asp) [29]. Any discrepancies were settled by all investigators through discussion.

### Statistical analysis

We conducted this meta-analysis based on the checklists and guidelines according to PRISMA [30]. The HWE was assessed for each study in the control groups using a Chi-square test, and every study with a calculated *P* less than 0.05 was considered a significant disequilibrium. ORs with 95% CIs were adopted to assess the strength of the relationship between the *HLA-G* 14 bp *Ins/Del* polymorphism and the risk of cancer in the homozygote comparisons (*Ins/Ins vs. Del/Del*), heterozygote comparisons (*Ins/Del vs. Del/Del*), dominant model (*Ins/Del + Ins/Ins vs. Del/Del*), recessive model (*Ins/Ins vs. Ins/Del + Del/Del*), and allelic comparisons (*Ins vs. Del*). Stratified analyses were carried out based on ethnicity (Asian, African, Caucasian, and Mixed population), type of cancer (publication with only one case–control study was merged as ‘other cancers’), and source of controls (hospital-based and population-based (PB)). Differences based on a Z-test were regarded as statistically significant if the *P*<0.05. The heterogeneity within each study was measured by a Cochran’s Q statistical test and the *I^2^* test [31]. A random-effects model was applied to measure the pooled OR when the *I^2^* value > 50%. Otherwise, a fixed-effects model was adopted according to the heterogeneity [32]. Sensitivity analysis was performed to assess the effect of each study on the pooled OR by removing each publication one by one to examine the stability of the overall results. Begg’s funnel plot test and Egger’s tests were applied to assess the potential publication bias [33,34]. All statistical analyses were conducted by STATA 12.0 software (version 12.0; STATA Corp. College Station, TX, U.S.A.). All of the tests were two-sided, and a *P*-value <0.05 was accepted as statistically significant.

## Results

### Characteristics of eligible studies

**Figure 1:**
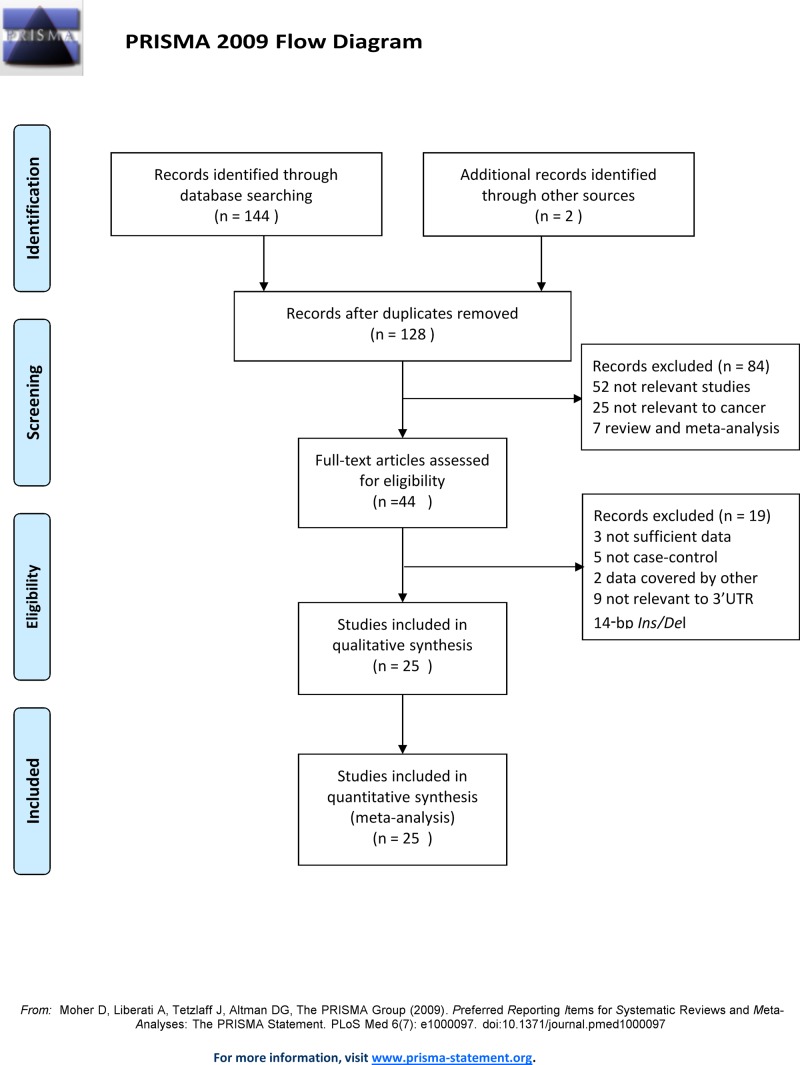
The flow diagram of the included and excluded studies

Figure 1 demonstrates the flow chart of the study selection process. After a systematic literature search in the databases mentioned above and a manual search in other sources, a total of 146 candidate articles were acquired. Eighteen search results were excluded as duplicates. Of the remaining 128 articles, 84 were removed after examining the titles and abstracts, resulting in a total of 44 articles. Among the 81 excluded studies, 52 were studies that were obviously irrelevant, 25 were not related to cancer, and 7 were reviews or meta-analyses. After carefully viewing the full text of the 44 potential studies to include in the meta-analysis, 19 of them were removed based on the following reasons: 3 did not have sufficient data, 5 were not case–control studies, 2 data were covered by other studies, and 9 were not relevant to the *HLA-G* 14 bp *Ins/Del* polymorphism. Finally, the remaining 25 eligible studies were included in the meta-analysis according to the inclusion and exclusion criteria [11,13,20–24,35–52]. A total of 4981 cases and 6391 controls are included in the current meta-analysis. The characteristics of the included case–control studies are displayed in Table 1. All studies were published between 2008 and 2018. With the exception of two publications reported in Chinese, all studies were written in English. Among all 25 studies, 10 studies were conducted in Asian populations, 7 in Caucasian populations, 6 in Mixed populations, and 2 in African populations. There were 11 different types of tumours in our study including: EC (*n*=2), non-small cell lung cancer (NSCLC) (*n*=2), breast cancer (*n*=5), cervical cancer (*n*=4), HCC (*n*=3), non-Hodgkin’s lymphoma (NHL) (*n*=2), thyroid cancer (*n*=2), prostate cancer (*n*=1), CRC (*n*=1), head and neck squamous cell carcinoma (HNSCC) (*n*=1), neuroblastoma (*n*=1), and bladder cancer (*n*=1). There were 12 PB studies and 13 hospital-based studies. All included studies used polymerase chain reaction (PCR) as the genotyping method with the exception of one study [39] that used DNA-PAGE. With the exception of one study [42], the genotype distributions of controls in all eligible studies did not deviate from the HWE. The distribution of genotypes and allele frequencies of the *HLA-G* 14 bp *Ins/Del* polymorphism in the cases and controls are provided in Table 2. Supplementary Table 1 demonstrated that the included studies were reliable based on the methodological quality.

**Table 1:** Characteristics of eligible case–control studies included in this meta-analysis

First author	Year	Country	Ethnicity	Cancer Type	Source of controls	Genotyping method	Number (case/control)	HWE	NOS score
Gao et al. [21]	2011	China	Asian	EC	HB	PCR	132/254	Yes	6
Xu et al. [35]	2017	China	Asian	NSCLC	PB	PCR	113/150	Yes	8
Zidi et al. [36]	2016	Tunisia	African	Breast cancer	PB	PCR	104/83	Yes	8
Zambra et al. [37]	2016	Brazil	Mixed	Prostate cancer	HB	PCR	187/129	Yes	7
Yang et al. [22]	2014	Taiwan	Asian	Cervical cancer	HB	PCR	315/400	Yes	7
Silva et al. [38]	2013	Brazil	Mixed	Cervical cancer	HB	PCR	55/50	Yes	7
Agnihotri et al. [39]	2017	India	Asian	HNSCC	PB	DNA-PAGE	383/383	Yes	8
Wisniewski et al. [40]	2015	Poland	Caucasian	NSCLC	PB	PCR	319/465	Yes	8
Teixeira et al. [41]	2013	Brazil	Mixed	HCC	PB	PCR	109/202	Yes	7
Haghi et al. [42]	2015	Iran	Asian	Breast cancer	PB	PCR	227/255	No	7
Garziera et al. [43]	2016	Italy	Caucasian	CRC	PB	PCR	308/294	Yes	8
Chen et al. [44]	2012	China	Asian	EC	HB	PCR	239/467	Yes	7
Tawfeek et al. [45]	2018	Egypt	African	NHL	PB	PCR	150/100	Yes	8
Dardano et al. [23]	2012	Italy	Caucasian	Thyroid cancer	HB	PCR	183/245	Yes	7
Ramos et al. [11]	2014	Brazil	Mixed	Breast cancer	HB	PCR	80/191	Yes	7
Eskandari-Nasab et al. [46]	2013	Iran	Asian	Breast cancer	PB	PCR	236/203	Yes	8
Lau et al. [24]	2011	Australia	Caucasian	Neuroblastoma	PB	PCR	153/404	Yes	8
Kim et al. [47]	2013	Korea	Asian	HCC	HB	PCR	270/91	Yes	7
Jiang et al. [13]	2011	China	Asian	HCC	PB	PCR	318/599	Yes	8
Jeong et al. [48]	2014	Korea	Asian	Breast cancer	HB	PCR	80/80	Yes	7
Ferguson et al. [49]	2012	Canada	Caucasian	Cervical cancer	HB	PCR	539/833	Yes	7
Bortolotti et al. [50]	2014	Italy	Caucasian	Cervical cancer	HB	PCR	100/100	Yes	7
Castelli et al. [20]	2008	Brazil	Mixed	Bladder cancer	PB	PCR	80/107	Yes	8
Bielska et al. [51]	2015	Poland	Caucasian	NHL	HB	PCR	207/150	Yes	7
de Figueiredo-Feitosa et al. [52]	2017	Brazil	Mixed	Thyroid cancer	PB	PCR	94/156	Yes	8

Abbreviation: HB, hospital-based.

**Table 2:** *HLA-G* 14–bp *Ins/Del* polymorphism genotype distribution and allele frequency in cases and controls

First author	Year	Genotype (*n*)								Allele frequency (*n*)				HWE
		Case				Control				Case		Control		
		Total	*Del/Del*	*Ins/Del*	*Ins/Ins*	*Total*	*Del/Del*	*Ins/Del*	*Ins/Ins*	*Del*	*Ins*	*Del*	*Ins*	
Gao et al. [21]	2011	132	54	66	12	254	77	128	46	174	90	282	220	0.852
Xu et al. [35]	2017	113	52	44	17	150	51	75	24	148	78	177	123	0.919
Zidi et al. [36]	2016	104	31	52	20	83	20	42	20	114	92	82	82	0.975
Zambra et al. [37]	2016	187	85	83	19	129	45	58	26	253	121	148	110	0.656
Yang et al. [22]	2014	315	169	110	36	400	188	176	36	448	182	552	248	0.850
Silva et al. [38]	2013	55	11	29	15	50	19	19	12	51	59	57	43	0.283
Agnihotri et al. [39]	2017	383	82	212	89	383	122	175	86	376	390	419	347	0.876
Wisniewski et al. [40]	2015	319	111	160	48	465	157	231	77	382	256	545	385	0.311
Teixeira et al. [41]	2013	109	49	44	16	202	70	87	45	142	76	227	177	0.205
Haghi et al. [42]	2015	227	56	127	44	255	52	154	49	239	215	258	252	0.004
Garziera et al. [43]	2016	308	97	138	73	294	114	122	58	332	284	350	238	0.059
Chen et al. [44]	2012	239	86	123	30	467	155	237	70	295	183	547	377	0.412
Tawfeek et al. [45]	2018	150	40	102	8	100	18	44	38	182	118	80	120	0.707
Dardano et al. [23]	2012	183	47	96	40	245	84	110	51	190	176	278	212	0.409
Ramos et al. [11]	2014	80	18	54	8	191	57	98	36	90	70	212	170	0.867
Eskandari-Nasab et al. [46]	2013	236	80	106	50	203	49	91	63	266	206	189	217	0.368
Lau et al. [24]	2011	153	66	58	29	404	146	194	64	190	116	486	322	0.973
Kim et al. [47]	2013	270	159	93	18	91	61	28	2	411	129	150	32	0.841
Jiang et al. [13]	2011	318	187	113	18	599	304	241	54	487	149	849	349	0.822
Jeong et al. [48]	2014	80	54	21	5	80	44	32	4	129	31	120	40	0.837
Ferguson et al. [49]	2012	539	184	242	113	833	272	399	162	610	468	943	723	0.770
Bortolotti et al. [50]	2014	100	49	40	11	100	38	40	22	138	62	116	84	0.201
Castelli et al. [20]	2008	80	28	37	15	107	35	50	22	93	67	120	94	0.868
Bielska et al. [51]	2015	207	49	91	67	150	33	89	28	189	225	155	145	0.071
de Figueiredo-Feitosa et al. [52]	2017	94	34	47	13	156	61	65	30	115	73	187	125	0.255

### Meta-analysis results

**Figure 2:**
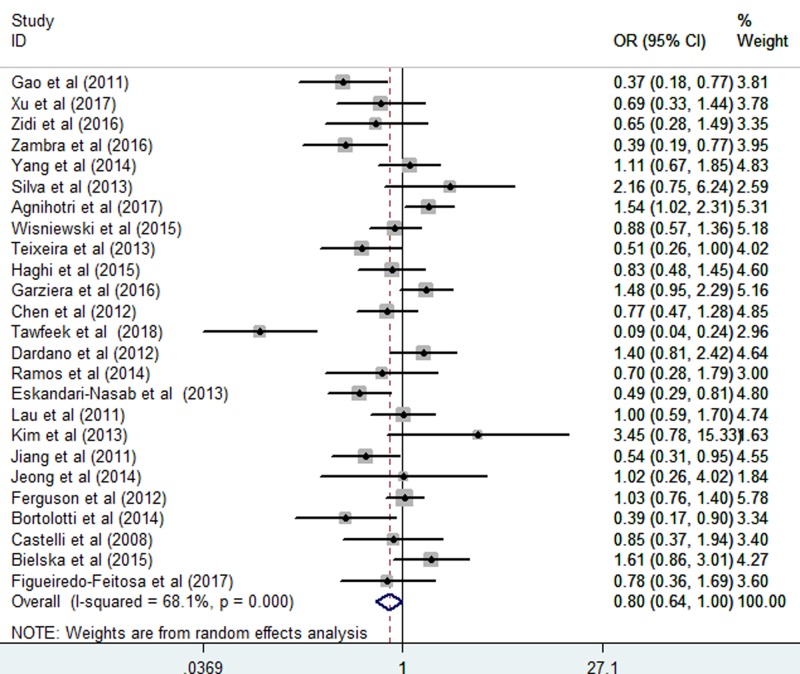
Forest plots of the *HLA-G* 14-bp *Ins/Del* polymorphism and cancer risk (homozygote comparisons: *Ins/Ins vs. Del/Del*)

**Figure 3:**
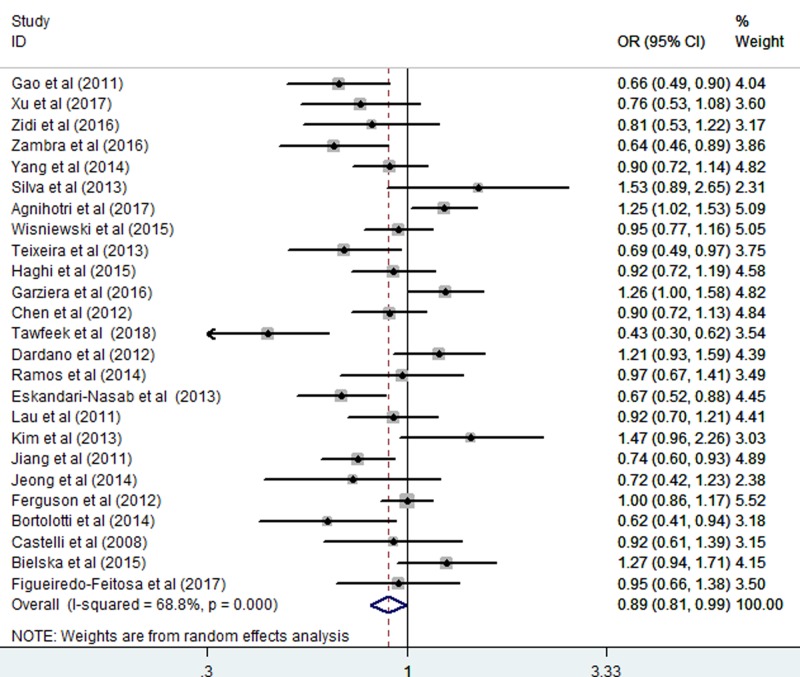
Forest plots of the *HLA-G* 14–bp *Ins/Del* polymorphism and cancer risk (allelic comparisons: *Ins vs. Del*)

The relationship between the *HLA-G* 14 bp *Ins/Del* polymorphism and cancer risk was assessed. The results revealed that the *HLA-G* 14 bp *Ins/Del* polymorphism was significantly associated with cancer risk in the homozygote comparison (*Ins/Ins vs. Del/Del*: OR = 0.80, CI = 0.64–1.00; *P*=0.049, Figure 2 and Table 3) and allelic comparison (*Ins vs. Del*: OR = 0.89, CI = 0.81–0.99; *P*=0.035, Figure 3 and Table 3). However, no significant association with cancer risk was found in other models including: *Ins/Del vs. Del/Del*: OR = 0.93, CI = 0.81–1.06; *P*=0.267; *Ins/Del + Ins/Ins vs.Del/Del*: OR = 0.82, CI = 0.68–1.01; *P*=0.056; and *Ins/Ins vs. Ins/Del + Del/Del*: OR = 0.89, CI = 0.78–1.02; *P*=0.107 (Table 3). The random-effects model was used due to the significant heterogeneity of the included studies.

**Table 3:** Analysis of the *HLA-G* 14 bp *Ins/Del* polymorphism and risk of cancer

Variables	*n*	Homozygote (Ins/Ins vs. Del/Del)		Heterozygote (Ins/Del vs. Del/Del)		Dominant (Ins/Del + Ins/Ins vs. Del/Del)		Recessive (Ins/Ins vs. Ins/Del + Del/Del)		Allelic (Ins vs. Del)	
		OR (95% CI)	*P_het_*	OR (95% CI)	*P_het_*	OR (95% CI)	*P_het_*	OR (95% CI)	*P_het_*	OR (95% CI)	*P_het_*
Total	25	**0.80 (0.64–1.00)**	0.000	0.93 (0.81–1.06)	0.000	0.82 (0.68–1.01)	0.000	0.89 (0.78–1.02)	0.000	**0.89 (0.81–0.99)**	0.000
Ethnicity											
Asian	10	0.81 (0.58–1.12)	0.003	0.84 (0.67–1.06)	0.001	0.87 (0.74–1.02)	0.068	0.83 (0.66–1.04)	0.000	0.87 (0.75–1.02)	0.001
African	2	0.25 (0.04–1.62)	0.003	0.92 (0.57–1.48)	0.585	0.26 (0.03–2.08)	0.000	0.67 (0.43–1.05)	0.641	0.59 (0.32–1.08)	0.026
Mixed	6	**0.67 (0.49–0.92)**	0.146	1.11 (0.77–1.59)	0.060	**0.64 (0.48–0.86)**	0.489	0.99 (0.69–1.43)	0.034	**0.85 (0.72–0.99)**	0.083
Caucasian	7	1.09 (0.91–1.29)	0.084	0.96 (0.77–1.19)	0.044	1.12 (0.87–1.43)	0.039	0.99 (0.81–1.21)	0.053	1.03 (0.90–1.19)	0.035
Type of cancer											
EC	2	0.56 (0.28–1.15)	0.104	0.86 (0.65–1.13)	0.409	**0.66 (0.45–0.96)**	0.156	0.80 (0.61–1.03)	0.223	**0.81 (0.67–0.97)**	0.118
NSCLC	2	0.83 (0.57–1.20)	0.583	0.79 (0.47–1.31)	0.094	0.90 (0.64–1.23)	0.918	0.80 (0.51–1.23)	0.124	0.90 (0.75–1.07)	0.288
Breast cancer	5	**0.65 (0.48–0.89)**	0.656	0.82 (0.65–1.05)	0.108	**0.74 (0.57–0.96)**	0.346	**0.77 (0.61–0.97)**	0.201	**0.82 (0.70–0.94)**	0.423
Other cancers	5	1.00 (0.64–1.58)	0.009	1.04 (0.70–1.56)	0.002	1.03 (0.84–1.26)	0.082	1.03 (0.70–1.52)	0.001	0.99 (0.78–1.26)	0.004
Cervical cancer	4	0.94 (0.62–1.54)	0.068	0.90 (0.64–1.26)	0.061	1.06 (0.85–1.32)	0.127	0.90 (0.65–1.24)	0.054	0.94 (0.74–1.18)	0.052
HCC	3	0.75 (0.34–1.65)	0.057	0.83 (0.67–1.04)	0.195	0.78 (0.40–1.53)	0.103	0.85 (0.56–1.30)	0.042	0.88 (0.59–1.31)	0.012
NHL	2	0.40 (0.03–6.47)	0.000	0.81 (0.53–1.22)	0.336	0.45 (0.02–9.65)	0.000	0.77 (0.52–1.14)	0.316	0.75 (0.26–2.15)	0.000
Thyroid cancer	2	1.15 (0.74–1.79)	0.223	1.26 (0.84–1.90)	0.616	0.92 (0.63–1.36)	0.291	1.35 (0.97–1.88)	0.407	1.11 (0.89–1.39)	0.294
Source of control											
HB	12	0.90 (0.68–1.23)	0.002	0.94 (0.77–1.14)	0.012	0.91 (0.67–1.23)	0.001	0.93 (0.77–1.12)	0.009	0.94 (0.81–1.09)	0.001
PB	13	**0.72 (0.53–0.99)**	0.000	0.92 (0.75–1.12)	0.003	**0.76 (0.58–1.00)**	0.000	0.86 (0.71–1.05)	0.000	**0.85 (0.73–0.99)**	0.000

Significant results (*P*<0.05) are highlighted in bold. Abbreviation: HB, hospital-based.

In the stratified analysis shown in Table 3, we explored the association between the *HLA-G* 14-bp *Ins/Del* variation and cancer risk in different ethnicities. The results showed a decreased cancer risk in Mixed populations based on three genetic models (*Ins/Ins vs. Del/Del*: OR = 0.67, CI = 0.49–0.92, *P*=0.014; *Ins/Del + Ins/Ins vs.Del/Del*: OR = 0.64, CI = 0.48–0.86, *P*=0.003; and *Ins vs. Del*: OR = 0.85, CI = 0.72–0.99, *P*=0.034). In a stratified analysis based on the cancer types, we found that the *HLA-G* 14-bp *Ins/Del* polymorphism was significantly associated with a reduced EC risk in the dominant model (*Ins/Del + Ins/Ins vs.Del/Del*: OR = 0.66, CI = 0.45–0.96, *P*=0.029) and in the allelic comparisons model (*Ins vs. Del*: OR = 0.81, CI = 0.67–0.97, *P*=0.022). Similar results were found in breast cancer based on all genetic models except for the heterozygote comparisons (*Ins/Ins vs. Del/Del*: OR = 0.65, CI = 0.48–0.89, *P*=0.007; *Ins/Del + Ins/Ins vs.Del/Del*: OR = 0.74, CI = 0.57–0.96, *P*=0.022; *Ins/Ins vs. Ins/Del + Del/Del*: OR = 0.77, CI = 0.61–0.97, *P*=0.024; and *Ins vs. Del*: OR = 0.82, CI = 0.70–0.94, *P*=0.006). In subgroups formed according to source of the controls, significantly decreased risks were observed in the PB analysis in the homozygote comparisons model (*Ins/Ins vs. Del/Del*: OR = 0.72, CI = 0.53–0.99, *P*=0.047), the dominant model (*Ins/Del + Ins/Ins vs.Del/Del*: OR = 0.76, CI = 0.58–1.00, *P*=0.048) and the allelic comparisons model (*Ins vs. Del*: OR = 0.85, CI = 0.73–0.99, *P*=0.040).

### Test of heterogeneity

A *Q* test and *I^2^* statistic were assessed to evaluate the heterogeneity among the selected studies. High heterogeneity was observed across studies, as well as in some subgroup analyses, as tested by random-effects analysis. Moreover, we evaluated the heterogeneity of all genetic models in regard to different ethnicities, cancer types, and the source of the controls. However, the observed heterogeneity could not be completely explained by different ethnicities, types of cancer, or the source of the controls (data not shown).

### Sensitivity analyses

**Figure 4:**
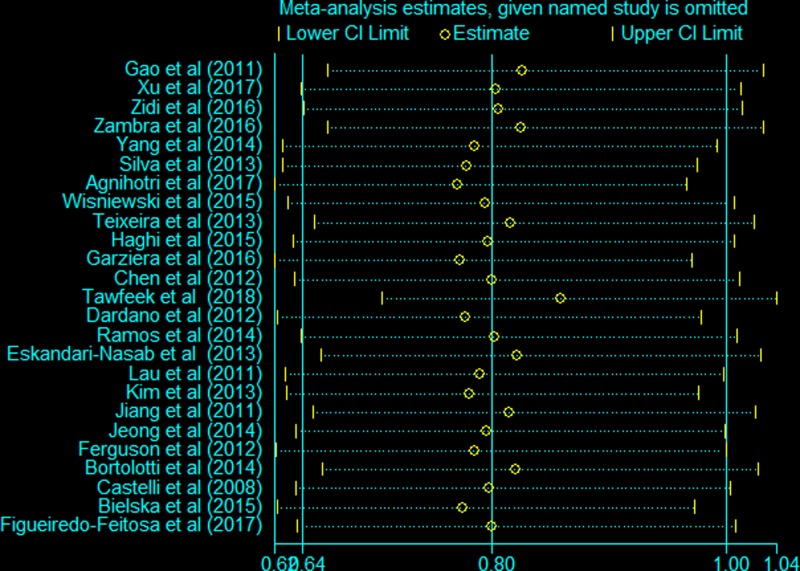
Sensitivity analysis of the *HLA-G* 14-bp *Ins/Del* polymorphism and cancer risk (homozygote comparisons: *Ins/Ins vs. Del/Del*)

Sensitivity analysis was carried out to examine the influence of each eligible study on the pooled ORs by the sequential removal of each individual study form the analysis. The individual removal procedure affected the pooled ORs, indicating the instability and unreliability of our findings for the homozygote comparisons (Figure 4). Sensitivity analyses of other genetic models yielded similar results (Supplementary Figure S1).

### Publication bias

**Figure 5:**
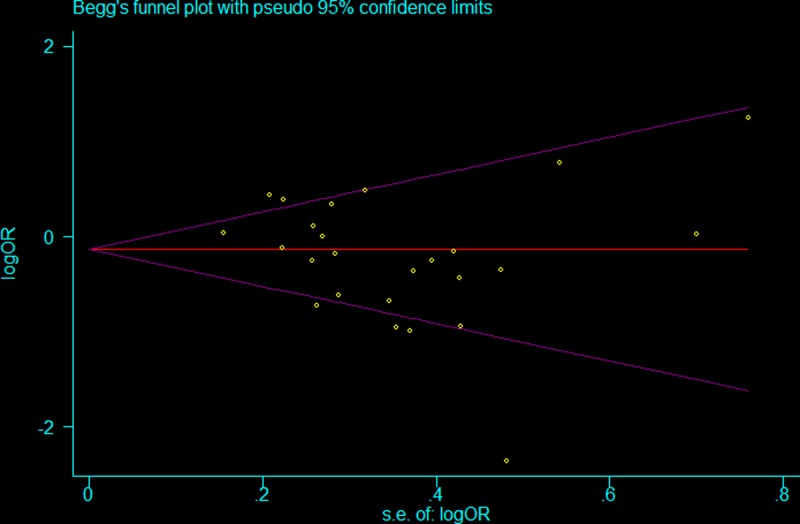
Funnel plot assessing evidence of publication bias (homozygote comparisons: *Ins/Ins vs. Del/Del*)

Begg’s and Egger’s tests were conducted to explore the potential for publication bias in assessment of the relationship between the *HLA-G* 14 *Ins/Del* polymorphism and cancer risk in all genetic models. No asymmetry was observed in the Begg’s funnel plots, and neither Begg’s rank correlation nor Egger’s regression showed publication bias among the studies (Figure 5 and Supplementary Table S2).

## Discussion

A well-characterised distinguishing feature of malignant tumours is their ability to evade antitumour immune destruction, which has proven to be a major contributor to tumorigenesis [53]. HLA-G is an important complex molecule that plays an important role in facilitating tumour escape from immune surveillance by its immunosuppressive function on T and NK cells [10], and the aberrant expression of HLA-G has been reported to be related to a variety of tumours [11–16]. The expression level of the HLA-G protein is related to *HLA-G* gene polymorphisms. The *Ins* allele has been shown to decrease the expression of HLA-G, and the *Del* allele has been shown to elevate the expression of HLA-G [19]. To date, a number of studies have explored the relationship between the *HLA-G* gene polymorphisms and the risk of cancer. Among the *HLA-G* gene polymorphisms, the *HLA-G* 14-bp *Ins/Del* polymorphism is the most widely explored. Up to now, multiple published case–control studies have investigated the underlying correlation between the *HLA-G* 14-bp *Ins/Del* polymorphism and cancer risk. However, the biological role of the *HLA-G* 14-bp *Ins/Del* polymorphism in the development of cancer remains poorly understood. Considering the inconsistent or even contradictory previously published results, and the fact that individual case–control studies may have been statistically underpowered, we assessed the effect of the polymorphism in the risk of cancer in the present meta-analysis. The present analysis includes all eligible studies to precisely explore the association of the *HLA-G* 14-bp *Ins/Del* polymorphism with cancer susceptibility.

In this meta-analysis, we evaluated the *HLA-G* 14-bp *Ins/Del* polymorphism and cancer risk relationship with all qualified case–control studies. In total, 4981 cases and 6391 controls were included. By quantificatively analysing the integrated data, the results of our present meta-analysis revealed that the *HLA-G* 14-bp *Ins/Del* polymorphism is significantly associated with the susceptibility of overall cancer. There were a larger number of studies that had evaluated the correlation between the *HLA-G* 14-bp *Ins/Del* polymorphism and the susceptibility to different types of cancer. However, the conclusions were paradoxical. Gao et al. [21] carried out a case–control study and found that the *HLA-G* 14-bp *Ins/Del* variant was associated with an elevated risk of EC. Similar results were found in other types of cancer, including thyroid cancer [52], breast cancer [46], and cervical cancer [22], among others. However, a few studies reported the opposite result, that the *HLA-G* 14-bp *Ins/Del* polymorphism could decrease the risk of some types of cancer. Additionally, some studies showed that the *HLA-G* 14-bp *Ins/Del* polymorphism did not play a role in cancer susceptibility. Furthermore, results from studies on the correlation between the *HLA-G* 14-bp *Ins/Del* polymorphism in the same types of cancer were inconsistent. For example, the study conducted by Teixeira et al. [41] demonstrated that individuals with the *HLA-G* 14-bp *Ins/Del* polymorphism had significantly increased risk for the occurrence of HCC, while Kim et al. [47] showed no relationship between the *HLA-G* 14-bp *Ins/Del* variant and HCC susceptibility; however, Jiang et al. [13] indicated that this variation may actually be a protective factor in HCC susceptibility. To address this controversy and to obtain a more accurate conclusion, several meta-analyses have been carried out several years ago [25–28]. Inconsistent with our present study, all of the previous meta-analyses reached the same conclusion: there was no relationship between the *HLA-G* 14-bp *Ins/Del* polymorphism and the risk of overall cancer. A latest meta-analysis of 21 published case–control studies with 3815 cases and 5802 controls was performed by Almeida et al. in 2018 [54]; however, they assessed the relationship between the *HLA-G* 14 bp *Ins/Del* polymorphism and the risk of cancer only in the allelic comparisons (*Ins vs. Del*), and no positive results were found. Our results demonstrated, for the first time, a significant relationship between the *HLA-G* 14-bp *Ins/Del* polymorphism and a decreased overall cancer risk. Compared with previous meta-analyses, our study included a larger sample size, a wider variety of cancer types, and a more diverse sample population. Hence, our results are persuasive based on their adequate statistical power.

Significant heterogeneity among the studies was shown in our results; we performed stratified analyses in terms of ethnicity, types of cancer, and sources of controls. In the subgroup analysis based on ethnicity, an obviously decreased cancer susceptibility was demonstrated in Mixed populations alone but not in Asian, African, or Caucasian populations. This discrepancy in cancer risk may be interpreted by geographic climate, daily lifestyle, ethnic diversity, dietary habits, as well as differences in alleles and genotypes in various ethnic populations. When carrying out stratified analysis by cancer type, we found that the *HLA-G* 14 bp *Ins/Del* polymorphism was significantly associated with a reduced EC and breast cancer risk, but we failed to find a significant risk association in other types of cancer. This result may be explained by the inherent heterogeneity of tumorigenic development in diverse cancer types [55]. Due to the relatively small sample size of each cancer type, inadequate statistical power may also be a factor in lacking a significant polymorphism–cancer risk relationship in these other cancer types. When we evaluated the *HLA-G* 14-bp *Ins/Del* polymorphism–cancer risk association according to source of the control, a significantly decreased risk was observed in PB controls but not in hospital-based controls; this result further verifies that the *HLA-G* 14-bp *Ins/Del* polymorphism is a potential protective factor for cancer. Previously, published meta-analyses also performed subgroup analysis to explore the association between the *HLA-G* 14 bp *Ins/Del* variant and risk of developing cancer; some significant results were reported and are partially in line with the conclusions from our present study. Zhang and Wang [25] conducted a meta-analysis in 2014 and found that the polymorphism was associated with risk of developing HCC in a subgroup analysis by cancer type. This finding was not in accordance with our result; however, only two case–control studies of HCC were included in their study. Li et al. [26] revealed a significant association between the *HLA-G* 14 bp *Ins/Del* variant and both breast cancer and PB control subgroup analyses, which is in agreement with the conclusions from our study. In 2015, Ge et al. [28] demonstrated the significant association in Asian populations and in breast cancer subgroups in stratified analyses. Inconsistent with their results, we found no association between the *HLA-G* 14 bp *Ins/Del* polymorphism and cancer risk in Asian populations in the present study. However, compared with their meta-analysis that included only six case–control studies on Asian populations, the results of our study, which involved ten case–control trials, have more adequate and more robust statistical power.

Despite our efforts to assess the association between the *HLA–G* 14–bp *Ins/Del* variant and the risk of cancer, there are several limitations we must account for in the present meta-analysis that may impact the objectivity of the findings. First, only unadjusted estimates were used to assess the strength of the relationship between the *HLA–G* 14–bp *Ins/Del* variant and the risk of developing cancer. The analysis cannot account for confounding factors such as life habit, environment factors, gene–gene interactions, gene–environment interactions, and even different variant loci in the same gene factors. Second, there may be a selection bias in our study, since only published case–control studies written in Chinese or English were included in our meta-analysis. Some potential eligible studies may have been excluded, because they were not detected, published, or because they were written in other languages. Third, although the total sample sizes of our meta-analysis were relatively large, the sample sizes of some stratified analyses were extremely small. There were not enough appropriate studies in some subgroups, weakening the statistical power to investigate the real relationship between the *HLA-G* 14-bp *Ins/Del* polymorphism and cancer risk. Fourth, because of the high heterogeneity in our present meta-analysis, the reliability of the findings may be weakened. Despite the application of the random-effects model in our meta-analysis, the findings on the overall cancer susceptibility should be taken cautiously. Fifth, the result of our meta-analysis should be interpreted with caution and needs to be confirmed by more case–control studies, because the sensitivity analyses indicated that deletion of certain individual study had an impact on the reliability of our results. Larger sample sizes and well-designed case–control experiments with various types of cancer in diverse ethnicities are needed to further verify the relationship between the *HLA–G* 14-bp *Ins/Del* variant and cancer risk.

In summary, the pooled results of our meta-analysis demonstrated that the *HLA–G* 14-bp *Ins/Del* polymorphism may play an important role in decreasing cancer susceptibility, especially in breast cancer and oesophageal cancer (EC), in the Mixed populations. The results allowed us to hypothesise that the *HLA–G* 14-bp *Ins/Del* variant may be a potential protective factor of cancer. Larger sample sizes and well-designed case–control experiments with various types of cancer in different ethnicities are needed to further verify our findings.

## Supporting information

**Supplementary Figure 1:** 

**Supplemental Table 1:** Methodological quality of the included studies according to the Newcastle-Ottawa Scale.

**Supplemental Table 2:** The results of Begg’s and Egger’s tests for the publication bias

## Abbreviations

CI: confidence interval
CRC: colorectal cancer
EC: oesophageal cancer/carcinoma
HCC: hepatocellular carcinoma
HLA: human leucocyte antigen
HWE: Hardy–Weinberg equilibrium
Ins/Del: insertion/deletion
NK: natural killer
OR: odds ratio
PB: population-based
3′UTR: 3′ untranslated region
