# Supplementary material for: Genetic variation in the HLA-G 3′UTR 14–bp insertion/deletion and the associated cancer risk: evidence from 25 case–control studies
Source: Biosci Rep. 2019 May 10;39(5):BSR20181991. doi: 10.1042/BSR20181991 (PMC6509057; doi:10.1042/BSR20181991)
Supplement: Supplementary file 1 [file bsr20181991_Supp1.pdf]

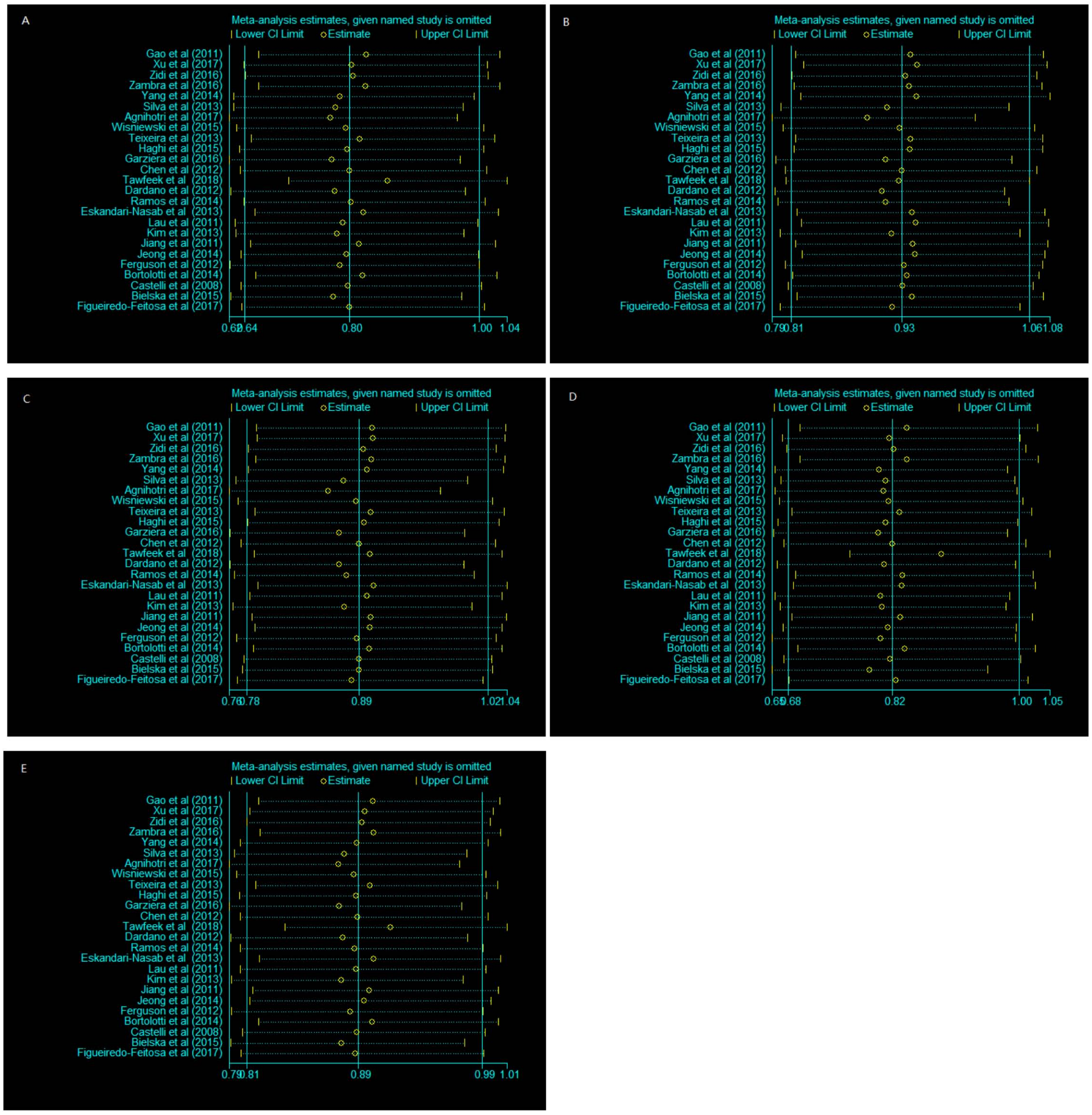
A
Meta-analysis estimates, given named study is omitted
Lower CI Limit
Estimate
Upper CI Limit
Gao et al (2011)
Xu et al (2017)
Zidi et al (2016)
Zambra et al (2016)
Yang et al (2014)
Silva et al (2013)
Agnihotri et al (2017)
Wisniewski et al (2015)
Teixeira et al (2013)
Haghi et al (2015)
Garziera et al (2016)
Chen et al (2012)
Tawfeek et al (2018)
Dardano et al (2012)
Ramos et al (2014)
Eskandari-Nasab et al (2013)
Lau et al (2011)
Kim et al (2013)
Jiang et al (2011)
Jeong et al (2014)
Ferguson et al (2012)
Bortolotti et al (2014)
Castelli et al (2008)
Bielska et al (2015)
Figueiredo-Feitosa et al (2017)
0.64
0.80
1.00
1.04
B
Meta-analysis estimates, given named study is omitted
Lower CI Limit
Estimate
Upper CI Limit
Gao et al (2011)
Xu et al (2017)
Zidi et al (2016)
Zambra et al (2016)
Yang et al (2014)
Silva et al (2013)
Agnihotri et al (2017)
Wisniewski et al (2015)
Teixeira et al (2013)
Haghi et al (2015)
Garziera et al (2016)
Chen et al (2012)
Tawfeek et al (2018)
Dardano et al (2012)
Ramos et al (2014)
Eskandari-Nasab et al (2013)
Lau et al (2011)
Kim et al (2013)
Jiang et al (2011)
Jeong et al (2014)
Ferguson et al (2012)
Bortolotti et al (2014)
Castelli et al (2008)
Bielska et al (2015)
Figueiredo-Feitosa et al (2017)
0.79
0.81
0.93
1.06
1.08
C
Meta-analysis estimates, given named study is omitted
Lower CI Limit
Estimate
Upper CI Limit
Gao et al (2011)
Xu et al (2017)
Zidi et al (2016)
Zambra et al (2016)
Yang et al (2014)
Silva et al (2013)
Agnihotri et al (2017)
Wisniewski et al (2015)
Teixeira et al (2013)
Haghi et al (2015)
Garziera et al (2016)
Chen et al (2012)
Tawfeek et al (2018)
Dardano et al (2012)
Ramos et al (2014)
Eskandari-Nasab et al (2013)
Lau et al (2011)
Kim et al (2013)
Jiang et al (2011)
Jeong et al (2014)
Ferguson et al (2012)
Bortolotti et al (2014)
Castelli et al (2008)
Bielska et al (2015)
Figueiredo-Feitosa et al (2017)
0.76
0.78
0.89
1.02
1.04
D
Meta-analysis estimates, given named study is omitted
Lower CI Limit
Estimate
Upper CI Limit
Gao et al (2011)
Xu et al (2017)
Zidi et al (2016)
Zambra et al (2016)
Yang et al (2014)
Silva et al (2013)
Agnihotri et al (2017)
Wisniewski et al (2015)
Teixeira et al (2013)
Haghi et al (2015)
Garziera et al (2016)
Chen et al (2012)
Tawfeek et al (2018)
Dardano et al (2012)
Ramos et al (2014)
Eskandari-Nasab et al (2013)
Lau et al (2011)
Kim et al (2013)
Jiang et al (2011)
Jeong et al (2014)
Ferguson et al (2012)
Bortolotti et al (2014)
Castelli et al (2008)
Bielska et al (2015)
Figueiredo-Feitosa et al (2017)
0.68
0.82
1.00
1.05
E
Meta-analysis estimates, given named study is omitted
Lower CI Limit
Estimate
Upper CI Limit
Gao et al (2011)
Xu et al (2017)
Zidi et al (2016)
Zambra et al (2016)
Yang et al (2014)
Silva et al (2013)
Agnihotri et al (2017)
Wisniewski et al (2015)
Teixeira et al (2013)
Haghi et al (2015)
Garziera et al (2016)
Chen et al (2012)
Tawfeek et al (2018)
Dardano et al (2012)
Ramos et al (2014)
Eskandari-Nasab et al (2013)
Lau et al (2011)
Kim et al (2013)
Jiang et al (2011)
Jeong et al (2014)
Ferguson et al (2012)
Bortolotti et al (2014)
Castelli et al (2008)
Bielska et al (2015)
Figueiredo-Feitosa et al (2017)
0.79
0.81
0.89
0.99
1.01

Supplemental Table 1 Methodological quality of the included studies according to the Newcastle-Ottawa Scale.

Study	Selection (score)				Comparability (score)	Exposure (score)			
	Adequate definition of patient case	Representativeness of patients cases	Selection of controls	Definition of control	Control for important factor or additional factor	Ascertainment of exposure	Same method of ascertainment for participants	Non-response Rate	Total Score
Gao et al [21]	*	*	NA	*	*	*	*	NA	6
Xu et al [35]	*	*	*	*	**	*	*	NA	8
Zidi et al [36]	*	*	*	*	**	*	*	NA	8
Zambra et al [37]	*	*	NA	*	**	*	*	NA	7
Yang et al [22]	*	*	NA	*	**	*	*	NA	7
Silva et al [38]	*	*	NA	*	**	*	*	NA	7
Agnihotri et al [39]	*	*	*	*	**	*	*	NA	8
Wisniewski et al [40]	*	*	*	*	**	*	*	NA	8
Teixeira et al [41]	*	*	*	*	*	*	*	NA	7
Haghi et al [42]	*	*	*	*	*	*	*	NA	7
Garziera et al [43]	*	*	*	*	**	*	*	NA	8
Chen et al [44]	*	*	NA	*	**	*	*	NA	7
Tawfeek et al [45]	*	*	*	*	**	*	*	NA	8
Dardano et al [23]	*	*	NA	*	**	*	*	NA	7
Ramos et al [11]	*	*	NA	*	**	*	*	NA	7
Eskandari-Nasab et al [46]	*	*	*	*	**	*	*	NA	8
Lau et al [24]	*	*	*	*	**	*	*	NA	8

Kim et al [47]	*	*	NA	*	**	*	*	NA	7
Jiang et al [13]	*	*	*	*	**	*	*	NA	8
Jeong et al [48]	*	*	NA	*	**	*	*	NA	7
Ferguson et al [49]	*	*	NA	*	**	*	*	NA	7
Bortolotti et al [50]	*	*	NA	*	**	*	*	NA	7
Castelli et al [20]	*	*	*	*	**	*	*	NA	8
Bielska et al [51]	*	*	NA	*	**	*	*	NA	7
Figueiredo-Feitosa et al [52]	*	*	*	*	**	*	*	NA	8

Supplemental table 2 The results of Begg’s and Egger’s tests for the publication bias

Comparison type	Publication bias	
	Begg’s test (*P*-value)	Egger’s tes(*P*-value)t
Ins/Ins vs. Del/Del	0.168	0.124
Ins/Del vs. Del/Del	0.559	0.760
Ins/Ins vs. Ins/Del + Del/Del	1.000	0.816
Ins/Del + Ins/Ins vs.Del/Del	0.168	0.088
Ins vs. Del	0.591	0.207
